# Novel Chromosomal Mutations Responsible for Fosfomycin Resistance in *Escherichia coli*

**DOI:** 10.3389/fmicb.2020.575031

**Published:** 2020-10-20

**Authors:** Vincent Cattoir, Annabelle Pourbaix, Mélanie Magnan, Françoise Chau, Victoire de Lastours, Brice Felden, Bruno Fantin, François Guérin

**Affiliations:** ^1^CHU de Rennes, Service de Bactériologie-Hygiène Hospitalière, Rennes, France; ^2^Centre National de Référence sur la Résistance aux Antibiotiques (laboratoire associé ‘Entérocoques’), Rennes, France; ^3^Inserm, Bacterial Regulatory RNAs and Medicine - UMR_S 1230, Rennes, France; ^4^IAME, UMR-1137, Inserm and Université de Paris Diderot, Paris, France; ^5^Service de Médecine Interne, Hôpital Beaujon, Assistance Publique – Hôpitaux de Paris, Paris, France; ^6^CHU de Caen, Service de Microbiologie, Caen, France; ^7^Université de Caen Normandie, EA4655, Caen, France

**Keywords:** *E. coli*, fosfomycin-resistant, *uhpB*, *uhpC*, *galU*, *lon*

## Abstract

Fosfomycin resistance in *Escherichia coli* results from chromosomal mutations or acquisition of plasmid-mediated genes. Because these mechanisms may be absent in some resistant isolates, we aimed at decipher the genetic basis of fosfomycin resistance in *E. coli*. Different groups of isolates were studied: fosfomycin-resistant mutants selected *in vitro* from *E. coli* CFT073 (MIC = 1 mg/L) and two groups (wildtype and non-wildtype) of *E. coli* clinical isolates. Single-nucleotide allelic replacement was performed to confirm the implication of novel mutations into resistance. Induction of *uhpT* expression by glucose-6-phosphate (G6P) was assessed by RT-qPCR. The genome of all clinical isolates was sequenced by MiSeq (Illumina). Two first-step mutants were obtained *in vitro* from CFT073 (MICs, 128 mg/L) with single mutations: G469R in *uhpB* (M3); F384L in *uhpC* (M4). Second-step mutants (MICs, 256 mg/L) presented additional mutations: R282V in *galU* (M7 from M3); Q558^∗^ in *lon* (M8 from M4). Introduction of *uhpB* or *uhpC* mutations by site-directed mutagenesis conferred a 128-fold increase in fosfomycin MICs, whereas single mutations in *galU* or *lon* were only responsible for a 2-fold increase. Also, these mutations abolished the induction of *uhpT* expression by G6P. All 14 fosfomycin-susceptible clinical isolates (MICs, 0.5–8 mg/L) were devoid of any mutation. At least one genetic change was detected in all but one fosfomycin-resistant clinical isolates (MICs, 32 – >256 mg/L) including 8, 17, 18, 5, and 8 in *uhpA*, *uhpB*, *uhpC*, *uhpT*, and *glpT* genes, respectively. In conclusion, novel mutations in *uhpB* and *uhpC* are associated with fosfomycin resistance in *E. coli* clinical isolates.

## Introduction

Fosfomycin, a phosphonic acid derivative discovered in 1969, has become the first-choice antibiotic for the ‘single-dose’ oral treatment of uncomplicated urinary tract infections (UTIs) (Falagas et al., 2016). It is a bactericidal antibiotic with a broad spectrum of activity that interferes with the first step of peptidoglycan synthesis in both Gram-positive and Gram-negative bacteria (Castaneda-Garcia et al., 2013; Falagas et al., 2016). As a phosphoenolpyruvate analog, fosfomycin inhibits the cytosolic UDP-*N*-acetylglucosamine enolpyruvyltransferase (also named MurA) by covalent binding to key residue C115 of the enzyme active site, preventing the formation of *N*-acetylmuramic acid (Kahan et al., 1974). This low-molecular-weight antibiotic enters into the bacterial cell via two transport uptake systems: the glycerol-3-phosphate permease (encoded by *glpT*) constitutively expressed and, the hexose phosphate uptake transporter (encoded by *uhpT*) inducible by extracellular glucose-6-phosphate (G6P) (Castaneda-Garcia et al., 2013). While transcription of *glpT* and *uhpT* is regulated by *glpR* and *uhpABC*, respectively, their expression also requires high levels of cyclic AMP (cAMP) combined with, as a complex, the cAMP receptor protein (CRP) (Castaneda-Garcia et al., 2013). cAMP levels depend on the activity of CyaA adenyl cyclase and are regulated by the phosphotransferase enzyme PtsI (Castaneda-Garcia et al., 2013).

Despite its widespread clinical use for many years in several countries, the prevalence of fosfomycin resistance is still low among *E. coli* clinical isolates, usually below 3% (4,5). Concerning multi-drug-resistant (MDR) isolates as ESBL-producing *E. coli*, levels of susceptibility to fosfomycin remain as high as 80% (Falagas et al., 2016, 2019; Aghamali et al., 2019). By contrast, the selection of fosfomycin-resistant mutants is much easier under *in vitro* conditions at high mutation frequencies (ca. 10^–8^–10^–7^) (Karageorgopoulos et al., 2012). This paradox is partially due to a significant resistance-associated fitness cost with decrease *in vitro* rate and attenuated virulence *in vivo* (Marchese et al., 2003; Nilsson et al., 2003; Pourbaix et al., 2017), and higher fosfomycin activity under urinary tract physiological conditions (i.e., urine acidification and anaerobiosis counterbalanced by negligible amounts of urinary G6P) that enhance expression of GlpT and UhpT (Martin-Gutierrez et al., 2018; Pourbaix et al., 2019).

Due to the unique mechanism of action of fosfomycin, there are no cross-resistances with other antibacterial agents (Falagas et al., 2016; Silver, 2017). However, three specific mechanisms of fosfomycin resistance were described in *E. coli*: impaired drug uptake, enzymatic drug inactivation and target modification (Cattoir and Guérin, 2018). Reduced drug uptake is the most frequent resistance mechanism for in *in vitro* mutants and clinical isolates. It results from chromosomal mutations that alter the function or expression of GlpT and/or UhpT transporters. These mutations (mutations, insertions, deletions) can arise either in structural genes (i.e., *glpT* and *uhpT*) or in genes coding for regulators (i.e., *uhpA*, *cyaA*, and *ptsI*) (Castaneda-Garcia et al., 2013; Silver, 2017). More recently, there is the emergence of plasmid-mediated metallo-dependent enzymes (including FosA, FosB, and FosX) that inactivate the drug, of which FosA3 is, by far, the most frequently variant in *E. coli* (Yang et al., 2019). Much more uncommon, fosfomycin resistance can be mediated by qualitative and/or quantitative modifications of MurA (Silver, 2017).

The aim of this study was to (1) investigate the genetic basis of fosfomycin resistance in *E. coli* mutants selected *in vitro* that had no mutations in genes previously reported to be involved in resistance (i.e., *glpT*, *uhpT*, *uhpA*, *murA*, *cyaA*, and *ptsI*), (2) demonstrate experimentally the role of novel mutations identified in four different genes, and (3) determine their prevalence among a collection fosfomycin-resistant *E. coli* clinical isolates recently collected in France.

## Materials and Methods

### Bacterial Strains

Three different groups of *E. coli* strains were used in this study (Tables 1, 2). The first group consisted of fosfomycin-resistant mutants (CFT073_M3 to CFT073_M8) obtained from the parental strain *E. coli* CFT073 (uropathogenic strain belonging to phylogroup B2) (Welch et al., 2002) after serial passages on Mueller–Hinton (MH) medium (Difco, Becton Dickinson, Rungis, France) containing increased concentrations of fosfomycin (from 32 to 128 mg/L) in the presence of G6P (25 mg/L). The two other groups consisted of *E. coli* epidemiologically unrelated clinical isolates (wildtype and non-wildtype phenotype of resistance to fosfomycin, according to the epidemiological cut-off established at 8 mg/L) responsible for UTIs in patients hospitalized in two French university hospitals between 2012 and 2017.

**TABLE 1:** Genotypic and phenotypic characteristics and susceptibility to fosfomycin of isogenic strains derived from *E. coli* CFT073.

**Strains**	**Characteristics**	**Fosfomycin MIC (mg/L)**	**Mutation(s) in:**									
			***uhpA***	***uhpB***	***uhpC***	***uhpT***	***glpT***	***murA***	***cyaA***	***ptsI***	***galU***	***lon***
*E. coli* CFT073	Wild-type susceptible strain (phylogenetic B2)	1	–	–	–	–	–	–	–	–	–	–
***In vitro* mutants**												
*E. coli* CFT073_M3	First-step resistant mutant derived from CFT073	128	–	G469R	–	–	–	–	–	–	–	–
*E. coli* CFT073_M4	First-step resistant mutant derived from CFT073	128	–	–	F384L	–	–	–	–	–	–	–
*E. coli* CFT073_M7	Second-step resistant mutant derived from CFT073_M3	256	–	G469R	–	–	–	–	–	–	R282V	–
*E. coli* CFT073_M8	Second-step resistant mutant derived from CFT073_M4	256	–	–	F384L	–	–	–	–	–	–	Q558*
**Knockout mutants and *trans*-complemented strains**												
*E. coli* CFT073 Δ*uhpT*	CFT073 derivative with complete deletion of *uhpT*	128	–	–	–	del^a^	–	–	–	–	–	–
*E. coli* CFT073 Δ*uhpB*	CFT073 derivative with complete deletion of *uhpB*	128	–	del	–	–	–	–	–	–	–	–
*E. coli* CFT073 Δ*uhpB*_pBAD202	CFT073 Δ*uhpB* carrying empty pBAD202 vector	128	–	del	–	–	–	–	–	–	–	–
*E. coli* CFT073 Δ*uhpB*_pBAD202-*uhpB*	CFT073 Δ*uhpB* carrying pBAD202Ω*uhpB*	1	–	–^b^	–	–	–	–	–	–	–	–
*E. coli* CFT073 Δ*uhpC*	CFT073 derivative with complete deletion of *uhpC*	128	–	–	del	–	–	–	–	–	–	–
*E. coli* CFT073 Δ*uhpC*_pBAD202	CFT073 Δ*uhpC* carrying empty pBAD202 vector	128	–	–	del	–	–	–	–	–	–	–
*E. coli* CFT073 Δ*uhpC*_pBAD202-*uhpC*	CFT073 Δ*uhpC* carrying pBAD202Ω*uhpC*	1	–	–^b^	–	–	–	–	–	–	–	–
*E. coli* CFT073 Δ*galU*	CFT073 derivative with complete deletion of *galU*	2	–	–	–	–	–	–	–	–	del	–
**Site-directed mutants**												
*E. coli* CFT073_*uhpB*^G469R^	CFT073 derivative with allelic replacement of *uhpB* by *uhpB*^G469R^	128	–	G469R	–	–	–	–	–	–	–	–
*E. coli* CFT073_*uhpC*^F384L^	CFT073 derivative with allelic replacement of *uhpC* by *uhpC*^F384L^	128	–	–	F384L	–	–	–	–	–	–	–
*E. coli* CFT073_*galU*^R282V^	CFT073 derivative with allelic replacement of *galU* by *galU*^R282V^	2	–	–	–	–	–	–	–	–	R282V	–
*E. coli* CFT073_*lon*^Q558^*	CFT073 derivative with allelic replacement of *lon* by *lon*^Q558^*	2	–	–	–	–	–	–	–	–	–	Q558*

*^*a*^del, deletion of the entire gene. ^*b*^Wildtype gene in multicopy.*

**TABLE 2:** Phenotypic and genotypic characteristics of clinical isolates.

**Strain**	**Phylogenetic group**	**β-lactam resistance phenotype^a^**	**Fosfomycin MIC (mg/L)**	**Mutation(s) in:**										**Presence of *fos* gene(s)**
				***uhpA***	***uhpB***	***uhpC***	***uhpT***	***glpT***	***murA***	***cyaA***	***ptsI***	***galU***	***lon***	
**Strains with an MIC of fosfomycin ≤ 8 mg/L^b^ (*n* = 14)**														
B60	B2	ESBL	1	–	–	–	–	–	–	–	–	–	–	–
B65	B2	ESBL	1	–	–	–	–	–	–	–	–	–	–	–
B69	B2	ESBL	8	–	–	–	–	–	–	–	–	–	–	–
B88	B2	ESBL	1	–	–	–	–	–	–	–	–	–	–	–
B108	B2	WT	2	–	–	–	–	–	–	–	–	–	–	–
B119	B2	WT	0.5	–	–	–	–	–	–	–	–	–	–	–
B120	B2	WT	1	–	–	–	–	–	–	–	–	–	–	–
B135	B2	WT	0.5	–	–	–	–	–	–	–	–	–	–	–
B140	B2	WT	0.5	–	–	–	–	–	–	–	–	–	–	–
B145	B2	WT	1	–	–	–	–	–	–	–	–	–	–	–
B151	B2	WT	1	–	–	–	–	–	–	–	–	–	–	–
C43	B2	PASE	1	–	–	–	–	–	–	–	–	–	–	–
C53	B2	PASE	2	–	–	–	–	–	–	–	–	–	–	–
C103	B1	ESBL	1	–	–	–	–	–	–	–	–	–	–	–
**Strains with an MIC of fosfomycin > 8 mg/L^b^ (*n* = 40)**														
B56	B2	ESBL	64	–	P169S	–	–	–	–	–	–	–	–	–
B97	B2	ESBL	64	–	–	G397D	–	–	–	–	–	–	–	–
B175	B2	WT	128	–	–	T72I	–	–	–	L125F	–	–	–	–
C05	B2	WT	128	–	–	Q210*	–	–	–	–	–	–	–	–
C06	E	ESBL	>256	Deleted operon				Q213*	–	–	–	–	–	–
C09	B1	PASE	256	–	T374S	–	–	C141Y	–	–	–	–	–	–
C10	D	PASE	256	–	–	1082_2557del		736_737insT	–	–	–	–	–	–
C20	B2	WT	128	281delG	T166I, P252S	–	–	–	–	–	R400H	–	–	–
C21	B2	WT	128	Deleted operon				–	–	–	–	–	–	–
C33	B2	WT	64	–	265_268del	–	–	–	–	–	–	–	–	–
C35	D	WT	256	S104*	D205A	–	–	–	–	–	–	–	–	–
C38	B2	HCASE	>256	–	–	–	–	P139Q	–	–	–	–	–	–
C41	D	PASE	256	–	A223V	Y18H	–	–	–	–	–	–	–	–
C44	D	PASE	64	A110S	D205A, A223V	G244D	–	–	–	–	–	–	–	–
C49	B1	PASE	32	–	W198*	I108M	–	–	–	–	–	–	–	–
C50	B2	PASE	64	–	H313Y	–	–	–	–	–	–	–	–	–
C51	B2	PASE	64	–	–	1068delT	–	Y223C	–	–	–	–	–	–
C55	A	WT	128	–	–	1068delT	–	Y223C	–	–	–	–	–	–
C62	D	WT	256	A110S	T166I, T374S	Q132*	–	–	–	–	–	–	–	–
C63	D	PASE	128	–	T166I, P252S	966_1239del	101_1392del	–	–	–	–	–	–	–
C64	B2	HCASE	64	–	Q60*	–	–	–	–	–	–	–	–	–
C68	D	PASE	128	–	Q76*	–	–	–	–	–	–	–	–	–
C73	D	PASE	>256	Deleted operon				–	–	–	–	–	–	–
C75	A	ESBL	128	R75C	–	–	–	–	–	–	–	–	–	–
C80	D	WT	128	–	P252S	G153S, G355S	–	–	–	–	–	–	–	–
C82	B1	WT	64	–	–	I108M	Y60F	–	–	–	–	–	–	–
C84	B1	WT	64	–	P36*	–	Q7*	–	–	–	–	–	–	–
C90	B1	WT	256	–	–	559_1105del	–	P97L	–	–	–	–	–	–
C91	D	PASE	128	Deleted operon				–	–	–	–	–	–	–
C93	B2	WT	256	–	–	459_532del	–	–	–	–	–	–	–	–
C98	B2	PASE	64	–	–	–	–	–	–	–	–	–	–	–
C100	B2	WT	>256	Deleted operon				–	–	–	–	–	–	–
C105	B2	WT	64	–	–	–	647_656del	–	–	–	–	–	–	–
C106	B1	HCASE	32	–	–	–	Q66*	Y223C	–	–	–	–	–	–
C110	B2	HCASE	128	A110S, 411_423del	–	A51S	–	–	–	–	–	–	–	–
C113	B2	PASE	128	120_129del	–	A51S	–	–	–	–	–	–	–	–
C114	B2	WT	256	–	–	Q132*	–	–	–	–	–	–	–	–
C115	B2	PASE	256	Q28*	–	–	–	–	–	–	–	–	–	–
C116	B2	PASE	>256	–	P218L	–	–	–	–	–	–	–	–	–
C127	B2	ESBL	256	–	P218L	–	–	–	–	–	–	–	–	–

*^*a*^ESBL, extended-spectrum β-lactamase; HCASE, hyperproduction of cephalosporinase; PASE, penicillinase; WT, wild-type. ^*b*^The epidemiological cut-off of fosfomycin in *E. coli* is 8 mg/L.*

Bacterial strains were routinely grown at 35°C in Luria–Bertani (LB) broth or agar supplemented with appropriate antibiotics, unless otherwise specified. When required, *E. coli* were grown on media supplemented with 100 mg/L ampicillin, 40 mg/L kanamycin or 25 mg/L chloramphenicol.

### Antimicrobial Susceptibility Testing

MICs of fosfomycin were determined by using the agar dilution reference method described by the European Committee on Antimicrobial Susceptibility Testing^1^. Briefly, bacterial suspension was prepared to match the turbidity of the 0.5 McFarland in sterile physiological water (ca. 10^8^ CFU/mL). Agar dilution was performed using MH agar plates containing 25 mg/L G6P. Cell suspensions were further diluted in MH broth and were delivered onto plates using a Steer replicator, which delivered ca. 10^4^ CFU for each isolate. Concentrations tested ranged from 256 to 0.125 mg/L. *E. coli* ATCC 25922 and *Pseudomonas aeruginosa* ATCC 25923 were used as control strains and were run in parallel with every experiment. Each MIC determination was performed at least three times. The current susceptibility breakpoint of fosfomycin for *Enterobacteriaceae* is a MIC ≤ 32 mg/L according to the EUCAST guidelines (see text footnote 1).

### *In vitro* Bacterial Growth Rate

Growth rates at 35°C were measured in Luria–Bertani (LB) broth and Nutrient broth (NB) at pH 5 or 7 as well as in sterile-filtered pooled human male urine (pH = 6.5). The bacteria were grown aerobically overnight at 35°C and approximately 10^5^ colony-forming units (CFUs) were inoculated into 200 μL of growth medium on a bioscreen plate and the optical density at 600 nm was read each 5 min for 24 h with a multimode reader Infinite 200 Pro^®^ (Tecan, Männendorf, Switzerland). Maximal growth rate (MGR) of each strain was calculated as the inflexion point of first by-product of the curve of growth. For each strain and condition, MGR was measured in duplicate in three separate experiments.

### Construction of the Knockout Mutants

The disruption of the genes coding for putative transporters (*glpT* and *uhpT*) and their regulators (*uhpA*, *uhpB*, and *uhpC*) were performed using the method previously described, with some modifications, using the Red helper plasmid pKOBEG (Datsenko and Wanner, 2000; Derbise et al., 2003). This vector is a low-copy-number plasmid that contains a gene for chloramphenicol resistance selection, a temperature-sensitive origin of replication, and a gene encoding a recombinase. Briefly, pKOBEG was first introduced into CFT073 competent cells by electroporation, and transformants were selected on LB agar with chloramphenicol (25 mg/L) after incubation for 24 h at 30°C. A selectable kanamycin resistance cassette (flanked by flippase recognition target [FRT] sequences) was amplified by PCR using DNA of pKD4 plasmid as the template. The primers used included 5′ extensions with homology for the candidate genes (around 50 bases) (Table 3). The PCR product was introduced into the pKOBEG-harboring CFT073 by electroporation, and after homologous recombination, the disruption of the candidate gene was obtained. Selected clones were cured for the pKOBEG plasmid following a heat shock, creating the kanamycin-resistant variant. In order to have deletion mutants free of the antibiotic marker, strains then were transformed with the pCP20_Gm plasmid, which is able to express the FLP nuclease that recognizes the FRT sequences present on either side of the *kan* gene (Doublet et al., 2008). Lastly, the mutants were verified by Sanger sequencing.

**TABLE 3:** Deoxynucleotide primers used in the study.

**Primer**	**Nucleotide sequence (5′ to 3′)**	**Purpose**
pKD4_uhpB_F	CTCCCGCTTAATTACCGTTATTGCCTGCTTTTTTATCTTCTCTGCCGGTGTAGGCTGGAGCTGCTTC	*uhpB* deletion
pKD4_uhpB_R	GAGGTAGAGAAACGCTGACACGCGTGCCGTGCAGACAGGAAATGGTCCATATGAATATCCTCCTTAG	
pKD4_uhpC_F	GTTGCCGTTTCTGAAAGCGCCTGCCGATGCGCCATTAATGACTGATAAGTGTAGGCTGGAGCTGCTTC	*uhpC* deletion
pKD4_uhpC_R	TCTCGCGGTGTCTGGGCGTTCAAAAAGGGCAGTAATAGCAGTGCGGAACATATGAATATCCTCCTTAG	
pKD4_uhpT_F	CCATGCTGGCTTTCTTAAACCAGGTTCGCAAGCCGACCCTGGACCTGTGTAGGCTGGAGCTGCTTC	*uhpT* deletion
pKD4_uhpT_R	AGTTACGTTTATGCCACTGTCAACTGCTGAATTTTTTTCTCGCGGCGGACATATGAATATCCTCCTTAG	
pKD4_galU_F	CGTTCAAAACACGAACAGTCCAGGAGAATTTAAATGGCTGCCATTAATAGTGTAGGCTGGAGCTGCTTC	*galU* deletion
pKD4_galU_R	CCGATACGGATGTTACTTCTTAATGCCCATCTCTTCTTCAAGCCAGGCTCATATGAATATCCTCCTTAG	
pDS132_F	CTGTTGCATGGGCATAAAGA	Verification of cloning in pDS132
pDS132_R	AGGAACACTTAACGGCTGAC	
Mut3/uhpB_G469R/xbaI-F1p	CCGTCTAGAGGTGGATTTATTGCTCTCGCTG	Site-directed mutagenesis for *uhpB*
Mut3/uhpB_G469R/*Xba*I-R1p	CCGTCTAGAGTGCGCCGCCGACGTTATGCGC	
uhpB_F	ACTGGGCGTCAGTAACGACG	Verification of *uhpB* sequence
uhpB_R	ATGGCGCATCGGCAGGCGCT	
Mut4/uhpC_F384L/Xba1-F1p	CCGTCTAGAGTATGGCGATCGTCGTGGGGA	Site-directed mutagenesis for *uhpC*
Mut4/uhpC_F384L/R1p	TCCCGTCGCCGCCCCTGCCGC	
Mut4/uhpC_F384L/F2p	*GCGGCAGGGGCGGCGACGGGA*TTGTCGGCTTGTTTGCTTATC	
Mut4/uhpC_F384L/*Xba*I-R2p	CCGTCTAGATACCCACGCCATAAGTGATG	
uhpC_F	TGTCTGCACGGCACGCGTGT	Verification of *uhpC* sequence
uhpC_R	GATAGCGTCCAGGCAAAACCT	
galU_R282V/xbaI-F1p	CCGTCTAGAGCCGCACGTGACTATTATGC	Site-directed mutagenesis for *galU*
galU_R282V/R1p	TACCGTATTCAACGAAGGCCTG	
galU_R282V/F2p	*CAGGCCTTCGTTGAATACGGTA*TCGTCATAACACCCTTGGCAC	
galU_R282V/*Xba*I-R2p	CCGTCTAGAGCGCAGGCAAGAGAATGTAC	
galU_F	TATACTGGGATGCGATACAG	Verification of *galU* sequence
galU_R	CACCGTTTCGTGGAAAACAC	
lon_Q558X/xbaI-F1p	CCGTCTAGATTAGCTGCGTTGTGCATATTG	Site-directed mutagenesis for *lon*
lon_Q558X_R1	GATGGTTTGCATCAGCGTCG	
lon_Q558X_F2	*CGACGCTGATGCAAACCATC*GAACGTATCGCACAAGCAT	
lon_Q558X/*Xba*I-R2p	CCGTCTAGA ACGACCATCAACCAGCACTT	
lon1_F	GCTTTCTACGTGTGCTGCAG	Verification of *lon* sequence
lon1_R	GCCATTCACGCTGCTGTAGCAT	
lon2_F	CCTTCGATGCCATTGAAGCTGA	
lon2_R	TTGAAGCACGCAGGATAGCT	
pBAB202_uhpB_F	CACCAAAACTGGCGCAAGGAATGG	Cloning of *uhpB* in pBAD202
pBAB202_uhpB_R	CAGAAACGGCAACATCATCG	
pBAB202_uhpC_F	CACCGCAACACGGTTTTGGCCTTA	Cloning of *uhpC* in pBAD202
pBAB202_uhpC_R	GATGCATCACGCTTCTCGC	
RT-qPCR_uhpT_F	ACCTACGGGTTGAGCATGAC	Quantification of *uhpT* expression
RT-qPCR_uhpT_R	CACTGAAGCCCAGCATACAA	
RT-qPCR_rrsA_F	CTCTTGCCATCGGATGTGCCCA	Quantification of *rrsA* expression^a^
RT-qPCR_rrsA_R	CCAGTGTGGCTGGTCATCCTCTCA	

*^*a*^Primers described in Peng et al., 2014.*

### Construction of *Trans*-Complemented Strains

The *uhpB* and *uhpC* wildtype genes were amplified by PCR using specific primers (Table 3) and each amplicon was TA cloned into the overexpression plasmid, pBAD202 directional TOPO (Invitrogen, Courtaboeuf, France). *E. coli* TOP10 cells (Invitrogen) carrying pBAD202 recombinants containing correctly oriented inserts were selected on LB plates with 40 mg/L kanamycin. After purification, recombinant plasmids pBAD202Ω*uhpB* and pBAD202Ω*uhpC* were used to transform by electroporation Δ*uhpB* and Δ*uhpC* mutants, respectively.

### Site-Directed Mutagenesis

Single-nucleotide allelic replacement was carried out using the suicide vector pDS132 in order to confirm the role of novel mutations (Philippe et al., 2004). The cloning steps of the desired gene alleles into pDS132 were performed in *E. coli* DH5αλ*pir* strain to allow replication of the plasmid. The recombinant plasmids were then purified and introduced in *E. coli* CFT073 by electro-transformation. The first step of allelic exchange was selection of plasmid integration into the recipient chromosome by plating cells on chloramphenicol-containing LB plates. After overnight growth at 35°C, one colony was picked, diluted in 10 mM MgSO_4_ solution, and serial dilutions were plated on LB agar plates with 5% sucrose and without NaCl. This plating step allowed selection of plasmid excision from the chromosome by a second cross-over. After overnight incubation at 35°C, 100 clones were streaked on chloramphenicol-containing LB agar plates and on LB agar with 5% sucrose and without NaCl. Several clones were screened by PCR-sequencing in order to identify those carrying the desired allele.

### RNA Extraction and RT-qPCR

The levels of expression of *uhpT* were determined by RT-qPCR using specific primers (Table 3). *E. coli* cells were grown for 24 h in LB broth, and the cells were harvested and washed twice with M9 minimum salt solution as previously described (Ohkoshi et al., 2017). The suspended cells were used to inoculate to M9 minimum salt solution with or without 0.2% G6P supplementation and incubated for 30 min at 35°C. Total RNAs were extracted from all clinical isolates using the Direct-zol RNA miniprep kit (Zymo Research, Irvine, CA, United States). Residual chromosomal DNA was removed by treating samples with the Turbo DNA-free kit (Life Technologies, Saint-Aubin, France). Samples were quantified using the BioSpec-nano spectrophotometer (Shimadzu, Noisiel, France), and the integrity was assessed using the Agilent 2100 bioanalyzer according to the manufacturer’s instructions. cDNA was synthesized from total RNA (∼25 ng) using the QuantiFast SYBR green RT-PCR kit (Qiagen), and transcript levels were determined by the ΔΔ threshold cycle (ΔΔCt) method using the rrsA (16S rRNA) gene as a housekeeping control gene (Table 3).

### WGS and Bioinformatic Analysis

Genomic DNA was isolated using the using the Quick-DNA fungal/bacterial miniprep kit (Zymo Research, Irvine, CA, United States). DNA libraries were prepared using the NEBNext Ultra DNA library prep kit for Illumina (New England Biolabs, Ipswich, MA, United States) and sequenced as paired-end reads (2 × 300 bp) using an Illumina MiSeq platform and the MiSeq reagent kit version 3. The Illumina reads were assembled using the CLC Genomics Workbench software (Qiagen). The annotation of chromosome and plasmids was performed using the NCBI Prokaryotic Genome Annotation Pipeline (PGAP)^2^. The nucleotide sequences were also submitted to ResFinder server^3^ (version 3.1) to identify fosfomycin resistance mutations and acquired genes. Raw and processed data generated in this study were deposited in GenBank as bioproject no. PRJNA625505.

## Results

### *In vitro* Fosfomycin-Resistant Mutants of *E. coli* CFT073

Four different mutants were selected *in vitro* from the parental strain *E. coli* CFT073, including two single-step and two second-step mutants (Table 1). The two first-step mutants harbored only one mutation each: CFT073_M3 possessed a non-synonymous mutation in *uhpB* (leading to the substitution G469R) and CFT073_M4 had a non-synonymous mutation in *uhpC* (leading to the substitution F384L). Both mutations were associated with a 128-fold increase in fosfomycin MICs (Table 1), and mutants were categorized as resistant according to the EUCAST breakpoints. Concerning the two-step mutants, CFT073_M7 and CFT073_M8 were obtained on agar plates supplemented with 128 mg/L of fosfomycin from CFT073_M3 and CFT073_M4, respectively. Both exhibited a two-fold increase in fosfomycin MICs (256 mg/L), and harbored one more mutation each: a non-synonymous mutation in *galU* (leading to the substitution R282V) in CFT073_M7, and a nonsense mutation in *lon* (leading to Q558^∗^) in CFT073_M8 (Table 1).

**FIGURE 1:**
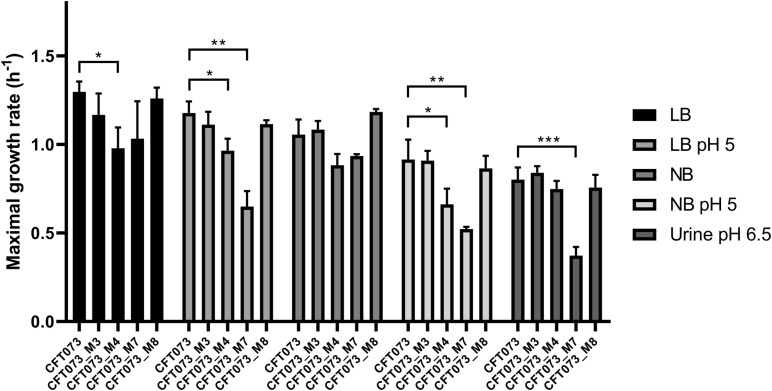
Maximal growth rates of CFT073 and *in vitro* mutants in Luria–Bertani (LB), LB pH 5, Nutrient broth (NB), NB pH 5 and urine (pH 6.5). Statistical comparison was performed using the unpaired *t*-test. *P < 0.05; **P < 0.01; ***P < 0.001.

Overall, bacterial growth rates were reduced as the pH was lower and exhibited their lowest levels in urine (Figure 1). MGR of CFT073_M4 was significantly decreased as compared to that of CFT073 (*P* < 0.05, unpaired *t* test), except in urine (Figure 1). Interestingly, CFT073_M7 had a significant decreased MGR as compared with CFT073 in LB and NB at pH 5 (*P* < 0.01, unpaired *t*-test) and also in urine at pH 6.5 (*P* < 0.001, unpaired *t* test) (Figure 1). There was no difference in MGRs for CFT073_M3 and CFT073_M8 (Figure 1).

### Role of the Novel Mutations Into Fosfomycin Resistance

To confirm the role of *uhpB*, *uhpC*, *galU*, and *lon* and their corresponding mutations in fosfomycin resistance, several approaches were used. First, knockout mutants were constructed, as well as their corresponding *trans*-complemented strains. Both Δ*uhpB* and Δ*uhpC* mutants were resistant to fosfomycin, with MICs at 128 mg/L (Table 1). As expected, the *trans*-complementation of Δ*uhpB* and Δ*uhpC* mutants with their respective isogenic copies restored the fosfomycin susceptibility, with MICs at 1 mg/L (Table 2). Whereas we failed to construct a Δ*lon* mutant, a deleted mutant was obtained for *galU* that only exhibited a two-fold increase in MICs of fosfomycin (Table 2).

Second, we constructed site-directed mutants of CFT073 by single-nucleotide allelic replacement, to introduce the same mutations than those observed in mutants obtained *in vitro* by antibiotic selection. The introduction of a unique mutation in *uhpB* (G469R) or in *uhpC* (F384L) was responsible for a significant increase in MICs (from 1 to 128 mg/L) in both cases (Table 1), confirming experimentally their role into fosfomycin resistance. The unique mutation in *galU* (R282V) conferred a two-fold increase in MIC of fosfomycin as did the sole mutation in *lon* (Q558^∗^) (Table 1). The latter results are consistent with the increase of MICs of fosfomycin in second-step mutants as compared to single-step mutants (256 vs. 128 mg/L, respectively).

**FIGURE 2:**
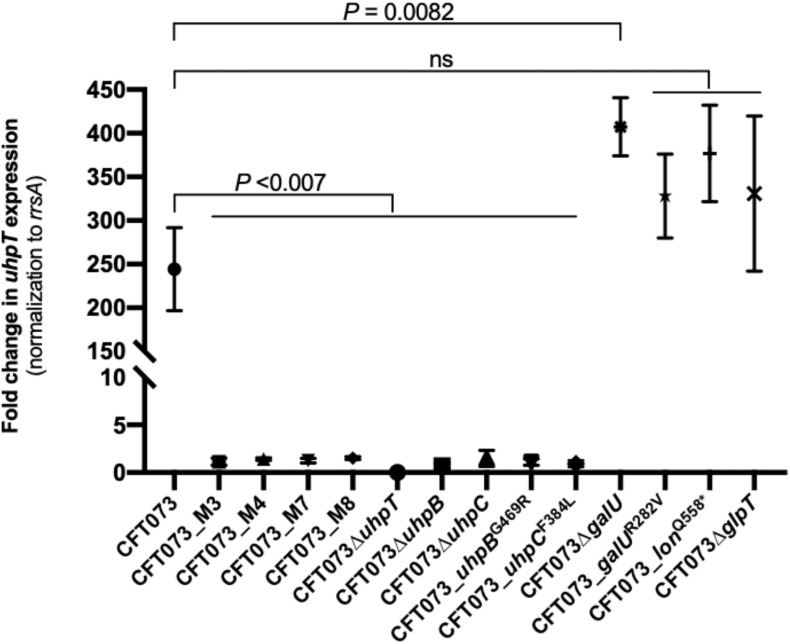
Changes in *uhpT* expression after induction with 0.2% of G6P in *E. coli* CFT073 parental strain and its derivative mutants. Transcript levels of *uhpT* are shown as relative values compared to those of *rrsA* (16S rRNA) gene. Data plotted correspond to the means and SDs of three biological replicates.

To understand the mechanism(s) by which these mutations confer higher fosfomycin MICs, we compared by RT-qPCR the differential expression of *uhpT* in the absence or presence of 0.2% G6P. After induction, *uhpT* expression was strongly enhanced (244-fold ± 47) in the CFT073 parental strain, as expected, whereas it was significantly lower in all mutants M3 (1.1-fold ± 0.4), M4 (1.4-fold ± 0.1), M7 (1.2-fold ± 0.2), and M8 (1.5-fold ± 0.1) (all *P* < 0.007 by an unpaired *t*) (Figure 2). This lack of induction by G6P was also observed with deleted and site-directed mutants for *uhpB* and *uhpC* (Figure 2). The deletion of *glpT* in CFT073 had no significant effect on G6P-mediated induction of *uhpT* expression, as expected, and it was also the case in CFT073_*galU*^*R*282*V*^ and CFT073_*lon*^Q558*^ mutants (Figure 2). Surprisingly, the change in *uhpT* expression after G6P induction was significantly higher in *galU*-deleted mutant than in the parental strain (407 ± 33 vs. 244-fold ± 47; *P* = 0.0082) (Figure 2).

### Prevalence of Novel Mutations in Fosfomycin-Resistant *E. coli* Clinical Isolates

To know if these mutations have been underestimated until now, we assessed their prevalence in a panel of 40 unrelated non-wildtype (MICs > 8 mg/L) *E. coli* clinical isolates (Table 2). We also studied a collection of 14 wildtype (MICs ≤ 8 mg/L) clinical isolates in which we verified the absence of mutations, as expected (Table 2).

Of the 40 non-wildtype isolates, no plasmid-mediated fosfomycin resistance genes (especially *fosA3*) were detected (Table 2). By contrast, at least one mutation/insertion/deletion was identified in almost all (*n* = 39) isolates, whereas only one isolate (C98, MIC at 64 mg/L) did not possess any change in *uhpA*, *uhpB*, *uhpC*, *uhpT*, *glpT*, *murA*, *cyaA*, *ptsI*, *galU* or *lon* genes (Table 2). Only two isolates (C49 and C106) were categorized as susceptible to fosfomycin (MICs at the susceptibility breakpoint, 32 mg/L) and harbored two mutations each (Table 2). Five isolates had a full deletion of the *uhp* operon, including one with one additional non-sense mutation in *glpT* (Q213^∗^). Besides these five cases, a genetic change was identified in *uhpA*, *uhpB*, *uhpC*, *uhpT*, *glpT*, *cyaA*, and *ptsI* in 8, 17, 18, 5, 8, 1, and 1 isolates, respectively (Table 2). Even though half of isolates presented several mutations in up to three genes, some unique mutations were sufficient to confer fosfomycin resistance (MICs ranging from 64 to >256 mg/L) such as in *uhpB* (Q60^∗^, Q76^∗^, 265_268del, P169S, P218L, and H313Y), *uhpC* (459_532del, Q132^∗^, Q210^∗^ and G397D), *uhpA* (Q28^∗^ and R75C), *uhpT* (647_656del), and *glpT* (P139Q) (Table 2). Finally, no mutations were detected in *galU* and *lon* genes among the 40 clinical isolates tested.

## Discussion

The Uhp hexose phosphate transport pathway and its regulation are well described in *E. coli* (Kadner, 1973; Kadner and Winkler, 1973; Kadner and Shattuck-Eidens, 1983; Weston and Kadner, 1987, 1988; Island et al., 1992; Island and Kadner, 1993; Wright et al., 2000; Verhamme et al., 2001, 2002). UhpT is a member of the Major Facilitator Superfamily (MFS) containing 12 transmembrane protein segments, and it is responsible for the accumulation of G6P into the bacterial cells. The UhpT system is tightly controlled by the UhpABC phosphorelay system UhpABC, which is necessary for high-level expression of *uhpT*. UhpC is also an MFS member that shares approximately 30% amino acid sequence identity with UhpT. UhpC is a membrane-bound protein that senses external G6P in the periplasm and interacts with UhpB, stimulating its kinase activity. UhpB is a membrane-bound histidine kinase (HK) in a two-component system that possesses eight predicted transmembrane helices and a C-terminal cytoplasmic domain containing the conserved sequence elements common to HK proteins (i.e., the H-box around the phosphorylated histidine, the N-box, and the G-box comprising the ATP-binding and phosphate transfer region) (Parkinson and Kofoid, 1992). Upon interaction with UhpC, UhpB autophosphorylates the conserved histidine residue (His313), with subsequent phosphorylation at Asp54 of its cognate response regulator UhpA. Phosphorylated UhpA increases the affinity for its specific DNA binding sites, hence promoting transcription of *uphT*.

Many mutants defective in the hexose phosphate transport were isolated between 1970s and 1990s, but shortcomings can be found in these old studies, such as the imprecise position of the mutation/deletion/insertion due to the poor annotation of the *uhp* region sequence, the absence of determination of fosfomycin MICs, and the ‘artificial nature’ of many *in vitro* mutants that were obtained by transposon insertion (Mu, Tn*10*), or a resistance cassette (Kadner, 1973; Kadner and Shattuck-Eidens, 1983; Weston and Kadner, 1987, 1988; Island et al., 1992). Also, mutations/insertions can have different impacts on fosfomycin susceptibility since some of them do not impair *uhpT* expression and others confer constitutive expression (Weston and Kadner, 1987; Island and Kadner, 1993). Deleted mutants with a kanamycin resistance cassette in *uhpA*, *uhpB*, or *uhpC* from the *E. coli* BW25113 parental strain only conferred a modest increase in fosfomycin MICs to 8, 8, and 4 mg/L (Castaneda-Garcia et al., 2009), respectively, which is different from our findings. Altogether, it suggests that ‘artificial’ insertional mutants do not represent systematically how bacteria develop fosfomycin resistance.

Unexpectedly, we found here novel mutations in *uhpB* and *uhpC* in mutants, which are not often detected in fosfomycin-resistant clinical isolates. Indeed, fosfomycin resistance in *E. coli* clinical isolates is usually due to chromosomal mutations in *uhpT*, *uhpA*, *glpT*, *murA*, *cyaA*, and *ptsI* genes (Nilsson et al., 2003; Oteo et al., 2009; Takahata et al., 2010; Li et al., 2015b; Tseng et al., 2015; Ohkoshi et al., 2017; Lucas et al., 2018; Seok et al., 2020), and little is known about the involvement of mutations in other genes, especially those in *uhpB* and *uhpC* that have been exceptionally reported (Castaneda-Garcia et al., 2013).

**FIGURE 3:**
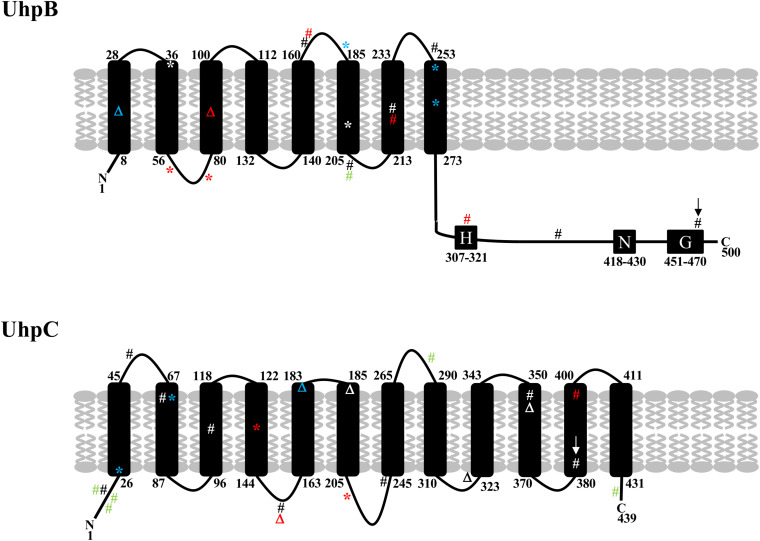
Schematic representation of the structure of UhpB and UhpC proteins with position of mutations identified in our study (in black and red) and previously described by Ballestero-Téllez (34) (in blue) and by Martin-Gutiérrez (10) (in green). Mutations in red correspond to single mutations associated with fosfomycin resistance in clinical isolates. Mutations with arrows correspond to single mutations (G469R in UhpB and F384L in UhpC) demonstrated experimentally to be responsible for fosfomycin resistance. Amino acids of transmembrane segments are indicated for UhpB (500 amino acids) and UhpC (439 amino acids). For UhpB, the putative conserved H-, N-, and G-boxes are also represented. #, non-synonymous mutation; *, non-sense mutation; Δ, deletion.

Recently, mutations in *uhpB* or *uhpC* were described in *E. coli* BW25133-derived laboratory mutants Δ*cyaA*, Δ*glpT-cyaA*, Δ*glpT-ptsI*, and Δ*ptsI-cyaA* recovered *in vitro* after time-kill experiments with fosfomycin (Ballestero-Tellez et al., 2017) and in two *E. coli* clinical isolates (Martin-Gutierrez et al., 2018). All the mutants were resistant to high levels to fosfomycin (MICs > 1,024 mg/L) and possessed the following mutations one or two mutations in *uhpB* (48del, W181^∗^, L255^∗^, and Q262^∗^) and *uhpC* (T27^∗^, T72P and 541_548del) (Ballestero-Tellez et al., 2017). In the two clinical isolates, one *uhpB* mutation (D205A) and three *uhpC* mutations (Y18H, G282D, T435A) were found in the first while two *uhpC* mutations (I14M, Q17Y) were found in the second (Martin-Gutierrez et al., 2018). We found here two mutations at the exact same position (D205A in UhpB and T72 in UhpC) of these previous studies (Figure 3), which is in favor of their role in fosfomycin resistance. In our study, *uhpB* mutations were distributed all along the 500-amino-acid-long protein in either periplasmic (*n* = 3), transmembrane (*n* = 5), or cytoplasmic (*n* = 6) regions, including one in the autophosphorylation H-box (H313Y) and another in the conserved G-box (G469R) that part of the ATP-binding domain (Figure 3). Concerning *uhpC* mutations, they were more frequently detected within the transmembrane segments (8/15) than into the cytoplasm (*n* = 6) or periplasm (*n* = 1) portions of the 439-amino-acid protein, suggesting that it could impair external G6P sensing through the membrane (Figure 3). Among fosfomycin-resistant clinical isolates, five had a full deletion of the *uhp* region (*uhpA*-*uhpB*-*uhpC*-*uhpT*), as reported (Lucas et al., 2018).

A majority of clinical isolates harbored *uhpB* and *uhpC* mutations that were widely distributed over the protein sequences. It is likely that all these mutations impact fosfomycin susceptibility differently, as described for insertion mutants exhibiting variable Uhp phenotypes (Weston and Kadner, 1988; Island et al., 1992; Island and Kadner, 1993). We also demonstrated that deletions and mutations in *uhpB* and *uhpC* were responsible for an absence of induction by G6P of *uhpT* expression, as described in several Δ*uhpA*, Δ*uhpB*, and Δ*uhpC* laboratory mutants and one clinical isolate with a truncated UhpA (Weston and Kadner, 1988; Island et al., 1992; Island and Kadner, 1993; Wright et al., 2000; Lucas et al., 2018). This confirms the role of UhpB and UhpC as G6P-response regulators required for the induction of *uhpT* expression. In addition, it appears that the mutation in *uhpC* (leading to the substitution F384L) also alters *in vitro* bacterial growth rate in LB and NB (regardless the pH) but not in urine, suggesting that it may occur *in vivo*.

Besides *uhpB* and *uhpC* mutations, two novel mutations were also identified in the two-step *in vitro* mutants. The first mutation occurred in *galU* that codes for a 302-amino-acid-long protein named UTP-glucose-1-phosphate uridylyltransferase, which catalyzes synthesis of UDP-D-glucose from UTP and α-D-glucose 1-phosphate (Weissborn et al., 1994). It is a central precursor for synthesis of cell surface carbohydrates, colanic acid, trehalose, cellulose, capsule- and membrane-derived oligosaccharides, and also has a major role in galactose metabolism (Ebrecht et al., 2015). Then, the deletion of *galU* has many consequences on different carbon metabolic pathways: for instance, they are unable to ferment galactose and fail to incorporate glucose and galactose into bacterial cell membranes, resulting in the incomplete synthesis of lipopolysaccharides (Fukasawa et al., 1962; Sundararajan et al., 1962). Also, the absence of *galU* leads to a reduced level of TolC into the outer membrane (Sharma et al., 2009), which might be related to antibiotic susceptibility. Here, we identified a non-synonymous mutation (R282V) in the C-terminal region of GalU that is outside the enzyme active site formed by the key residues T20, R21, and K202 (Ebrecht et al., 2015). Then, it is difficult to explain the implication of R282V mutation into the fosfomycin MIC two-fold increase. Note that it seems that this mutation also impacts on bacterial fitness when grown in acidic pH or in urine, suggesting that it may be difficult to develop *in vivo*.

The second mutation appeared in *lon* coding for an ATP-dependent serine protease that plays a major role in protein quality control, degrading incorrect proteins, and has an important role into many biological processes in bacteria (Tsilibaris et al., 2006). It degrades abnormal and misfolded proteins, but has also regulatory proteins as substrates, such as MarA and SoxS (Griffith et al., 2004). Here, we identified a *lon* mutation giving rise to a premature stop codon (Q558^∗^), and then a truncated protein, probably not functional. Indeed, with a length of 784 amino acids in *E. coli*, a large part of the C-terminal domain is lacking (Amerik et al., 1991). Therefore, we can assume that this truncated protein is inactive since the Ser679-Lys722 catalytic dyad is absent (Botos et al., 2004). Interestingly, it was demonstrated that mutations in *lon* were implicated in the development of multiple antibiotic resistance phenotype related to the efflux pump system AcrAB-TolC, and to the OmpF porin (Nicoloff et al., 2006, 2007; Duval et al., 2009; Nicoloff and Andersson, 2013; Bhaskarla et al., 2016). MarA, SoxS, and Rob, positively control the expression of *acrAB*, *tolC*, and *micF*, and *micF* regulatory RNA post-transcriptionally represses the translation of *ompF* mRNA (Li et al., 2015a). In a *lon* mutant, the accumulation of MarA and SoxS could enhance the *micF*-mediated inhibition of the OmpF production, that could impact on fosfomycin activity since OmpF can facilitate the spontaneous diffusion of the antibiotic across the outer membrane (Golla et al., 2019).

In conclusion, we demonstrated here experimentally the role of novel mutations in four genes implicated in fosfomycin resistance, and the prevalence of *uhpB* and *uhpC* mutations among fosfomycin-resistant *E. coli* clinical isolates.

## Data Availability Statement

The datasets presented in this study can be found in online repositories. The names of the repository/repositories and accession number(s) can be found in the article/Supplementary Material.

## Author Contributions

VC and FG conceptualized the study. VC, AP, MM, FC, VL, BF, and FG contributed to methodology. VC and FG provided the formal analysis and visualization. VC, AP, MM, FC, VL, BF, BF, and FG carried out the investigation. VC, BF, and FG were responsible for the resources. VC, BF, BF, and FG wrote the manuscript. All authors read and approved the manuscript.

## Conflict of Interest

The authors declare that the research was conducted in the absence of any commercial or financial relationships that could be construed as a potential conflict of interest.

## Footnotes

1 www.eucast.org

2 www.ncbi.nlm.nih.gov/genome/annotation_prok/

3 https://cge.cbs.dtu.dk/services/ResFinder/
